# Red Swamp Crayfish (*Procambarus clarkii*) as a Growing Food Source: Opportunities and Challenges in Comprehensive Research and Utilization

**DOI:** 10.3390/foods13233780

**Published:** 2024-11-25

**Authors:** Bimin Chen, Xiaoqi Xu, Yinji Chen, Hongkai Xie, Tao Zhang, Xiangzhao Mao

**Affiliations:** 1 College of Food Science and Engineering, Nanjing University of Finance and Economics/Collaborative Innovation Center for Modern Grain Circulation and Safety, Nanjing 210023, China; chenbmin@126.com (B.C.); chenyinji@nufe.edu.cn (Y.C.); xiehongkai@nufe.edu.cn (H.X.); 2 College of Food and Light Industry, Nanjing Tech University, Nanjing 211816, China; xiaoqi_xu@njtech.edu.cn; 3 College of Food Science and Engineering, Ocean University of China, Qingdao 266003, China; xzhmao@ouc.edu.cn

**Keywords:** *Procambarus clarkii*, nutrient composition, safety evaluation, processing, utilization

## Abstract

The red swamp crayfish (*Procambarus clarkii*) was introduced from Japan to China in the 1920s. Crayfish are now widely distributed in almost all types of freshwater wetlands, including rice fields, ditches, swamps, lakes, and ponds in most provinces of China, owing to their multi-directional movement, rapid growth, adaptability to the environment, and relatively high fecundity. The delectable taste and high nutritional value of crayfish have made them popular among consumers, leading to the significant development of red swamp crayfish farming in the last two decades. Currently, it represents the largest proportion of commercially farmed freshwater crustaceans in China and has become an integral component of China’s aquatic economy. Crayfish are highly valued for their edibility and for their by-products, which have various important uses. This review discusses nutrient composition, active ingredients, safety evaluation, processing and preservation, and comprehensive utilization of crayfish by-products to explore and organize the existing knowledge about crayfish and to promote the growth of the crayfish industry. This comprehensive review aims to provide a basis for the optimal utilization and sustainable development of crayfish resources worldwide.

## 1. Introduction

**Figure 1:**
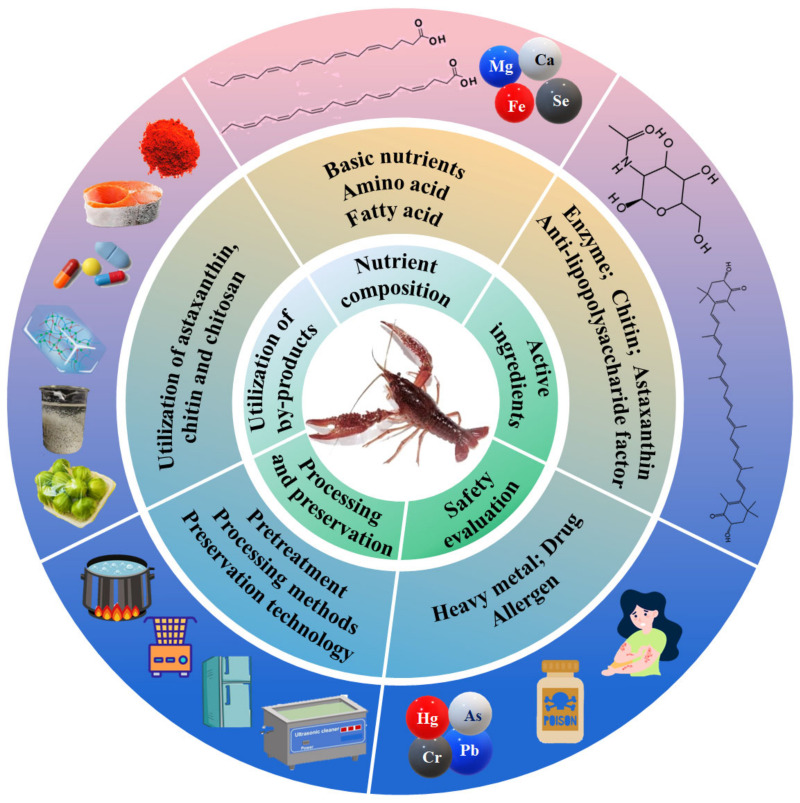
The main contents of this review on red swamp crayfish (*Procambarus clarkii*).

The red swamp crayfish (*Procambarus clarkii*) is native to northern Mexico and the southern United States. Their accessibility and nutritional value have contributed to make crayfish a relevant food item for many societies and a source of economic development, which has been reported in many literatures in various countries from different professional perspectives, as it is now one of the most relatively well-known species and exploited by humans in many regions around the globe [1]. The use of crayfish as food is in the roots of several cultural traditions, such as the Swedish crayfish summer festivals and Xuyi crayfish festival in China, in which families and friends gather to eat crayfish [1]. It was introduced to China from Japan in the 1920s and has become an important part of China’s aquatic industrial economy. According to estimates, the total output value of China’s crayfish industry was USD 64.07 billion in 2022, with an annual growth of 7.99%, which is higher than the level in 2021 (USD 59.33 billion). In 2022, the area of crayfish farming in China reached 18.67 million hectares, and the output reached 2.89 million tons, which was up by 7.69% and 9.76% annually, respectively. Crayfish farming accounts for 8.79% of the total freshwater aquaculture production in China, with an annual increase of 0.51%. Now, crayfish farming production has ranked fourth among freshwater aquaculture varieties in China. In addition to being delicious, crayfish have high nutritional value and are rich in protein, amino acids, and unsaturated fatty acids. The by-products of crayfish processing (such as heads and shells) also contain many useful ingredients, including proteins, lipids, minerals, and pigment that can be applied in a variety of fields [1]. This review summarized the nutritional composition, active ingredients, processing and preservation, comprehensive utilization of by-products, and safety evaluation of crayfish, and discusses the opportunities and challenges faced by the crayfish industry, aiming to improve the effective use of these resources, enhance market competitiveness, and promote the development of the crayfish industry (Figure 1).

## 2. Nutrient Composition

The chemical composition and nutritive values of fish and shellfish vary depending on several factors, including species variety and nutritive degree, diet, season of harvesting, location of capture, and environmental conditions [2,3]. The basic nutrients in crayfish are shown in Table 1.

### 2.1. Basic Nutrients

Moisture, protein, ash, and fat are the basic nutrients in muscles, and their contents are important indicators to evaluate muscle quality. The average moisture, ash, crude protein, and crude fat contents in dry crayfish (*P. clarkia*) abdominal samples, for example, from the River Nile, Egypt, are 75.75 ± 2.3%, 9.3 ± 3.9%, 73.25 ± 4.1%, and 7.564 ± 0.6%, respectively [8]. Feed intake affects essential nutrient composition in crayfish. Hepatopancreatic crude lipid and ash content were significantly higher in the biofloc group than in the diet group when red swamp crayfish were fed a commercial diet and Biofloc technology (*p* < 0.05) [6]. Crayfish muscle crude protein content increases from 20.70% to 41.12% with increasing dietary protein levels, and significantly declines when dietary protein increases to 44.64% [9]. The crayfish from different living environments also had a distinct nutritional quality. The flesh of the rice field crayfish had the highest moisture content (81.43%) and the lowest protein content (15.50%), and higher lipid content was found in the flesh of the rice field crayfish (1.10%) and the flesh of the pond crayfish (1.19%) [10]. Crayfish are rich in phosphorus, magnesium, calcium, and other important minerals, of which magnesium may play an important role in the prevention of cardiovascular diseases such as hypertension. Crayfish also contain selenium, zinc, iron, copper, and other trace elements. The retinol equivalent is extremely rich, and selenium and retinol are beneficial for individuals with weak vision [7,11,12].

### 2.2. Amino Acids

Crayfish from the Nile River contain seven amino acids. Two of them are non-essential amino acids (NEAAs) and five are essential amino acids (EAAs). The EAA values for isoleucine, leucine, lysine, methionine, and valine were 0.529, 0.131, 0.181, 0.016, and 0.058 mg/g, respectively [8]. Zaglol and Eltadawy [7] detected nine EAAs and eight NEAAs in crayfish proteins. The most abundant amino acids are glutamic acid, aspartic acid, and arginine, and crayfish contain EAAs including arginine (which is less abundant in vertebrates) and histidine, which is essential for children. Due to many differences in the growing environment and food composition, the total amino acid content of crayfish in different culture modes was different. It was 16.21 g/100 g in pond crayfish meat, and 12.74 g/100 g in rice field ones [10]. When comparing the ratio of crayfish protein to that of farmed, fresh, and frozen shrimp, and other proteins of high biological value, such as beef, eggs, and milk, it revealed little difference in amino acid levels and no significant reduction in nutritional value [11], as shown in Table 2.

### 2.3. Fatty Acids

The fat content is relatively low and mainly contains USFAs, as different crayfish samples show in Table 3, which are conducive to human digestion and absorption. The fatty acid profile of crayfish from the Nile River, Egypt, show eight saturated fatty acids (SFAs) and seven unsaturated fatty acids (USFAs). The total content of SFA in the lipids is 31.1% and the total content of USFA is 38.29%. The predominant SFA is palmitic acid (C16:0; 26.63%), whereas the predominant USFA is oleic acid (C18:1n-9; 29.26%) [7]. Crayfish contain high levels of USFAs that are conducive to digestion and absorption and are a good source of ω-3 and ω-6 fatty acids, essential for brain development, body building, and pregnancy.

## 3. Active Ingredients

Crayfish contain active ingredients with medicinal utility or physiological activity, such as enzymes, chitin, and astaxanthin. Chitinase degrades chitin and plays an essential role in the immunity of animals and the defense of plants. PcChitinase 2 may be involved in the innate immune response of crayfish through the modulation of the Toll pathway [14]. Another enzyme named prophenoloxidase (proPO) was also found in crayfish, which is located in the cytoplasm of hemocytes with a critical role in the antibacterial innate immune response, and is involved in multiple physiological processes, such as melanization, cytotoxic reactant production, particle encapsulation, and hemocyte attraction, inducing phagocytosis, and the formation of nodules and capsules [15]. Superoxide dismutases (SODs) are important antioxidant enzymes that remove excess amounts of biologically reactive oxygen intermediates. The SODs in crayfish play an important role in the innate immune responses against *Spiroplasma eriocheiris* and *Aeromonas hydrophila* [16].

**Figure 2:**
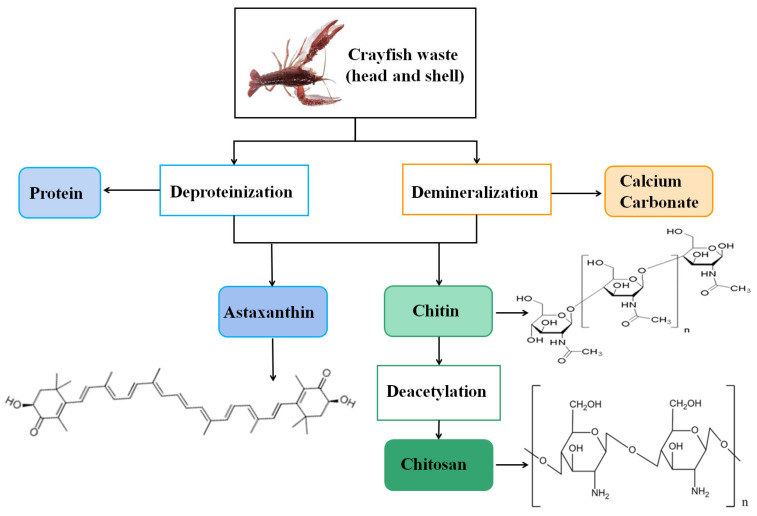
The typical utilization of crayfish waste with the chemical structures of three main by-products with bioactivities.

It was reported that crayfish shells contain approximately 20–40% of chitin [17]. Chitin is an organic polymer compound, the second most abundant in nature after cellulose, and the only natural alkaline polysaccharide discovered so far [18,19]. Chitin is chemically stable, insoluble, and can be deacetylated to form chitosan (Figure 2). Chitin is the primary product of the comprehensive utilization of freshwater crayfish and is widely used in daily chemicals, medicines, and food processing [20]. Due to its biodegradability, biocompatibility, and renewability, the efficient utilization and degradation of this biomass resource has recently attracted intense research interest [21]. It is a natural polymer material that is used as a wound-healing promoter, drug delivery carrier, and surgical suture with broad utility in biomedicine [22]. As a major fishing nation, China ranks first in the world in terms of production of aquatic products and has experienced explosive growth over the past decade, resulting in a large amount of shellfish waste; therefore, hydrolysis of crayfish shells and further access to their hydrolysis products can solve the pollution of chitin waste and improve the use of the by-products with a high added value. There have been extensive literature reports on how to extract chitin from crayfish and its further processing and utilization, which would be discussed in the following section.

Astaxanthin is a type of carotenoid found mainly in aquatic animals and is responsible for the unique red color of crustaceans. It is also used as a natural pigment and can be extracted from algae, yeast, and shellfish by-products, including crayfish shells [23]. It has high antioxidant activity due to its unique structure, which includes keto (C=O) and hydroxyl (OH) endings to donate hydrogen, and has a strong scavenging effect on free radicals and can act as an antioxidant, enhance immunity, prevent cancer, and improve human health through ultraviolet (UV) protection and anti-inflammatory activity [24,25]. Additionally, it can protect the central nervous and visual systems [26,27]. Moreover, as a therapeutic agent for a wide range of diseases without toxicity or side effects, it is an effective anti-tumor agent due to its ability to prevent the migration of cancer cells, anti-apoptosis, and anti-proliferation, and its involvement in the general support of the immune system [28].

The lack of an adaptive immune system in crustaceans such as crayfish has led to the evolution of effective molecules such as antimicrobial peptides, which are used to defend against pathogenic microbes, which might inspire researchers to develop natural products rather than synthetic drugs for health or medical purposes. Anti-lipopolysaccharide factors (ALF) are a group of innate immunity effector molecules in arthropods that bind and neutralize lipopolysaccharides, and represent one of the most evolutionarily conserved cationic anti-microbial peptides broadly distributed among crustaceans. Sun presented the identification and characterization of an ALF from *P. clarkii* (*PcALF1*), and demonstrated a broad spectrum against Gram-positive and Gram-negative bacteria through the antimicrobial activity assay in vitro [29]. Another new one from *Procambarus clarkii* (*PcALF*) showed a high level of transcription against the microbial pathogens administered, with a significant up-regulation against these pathogens being observed. PcALF may play a critical biological role in crayfish immune defense, suggesting a potential therapeutic agent for disease control and health management, based on its tissue expression patterns and responses to viral and bacterial challenges [30]. It should be noted that only a few studies have described the ALF immune responses in invertebrates, especially crustaceans.

## 4. Processing and Preservation of Crayfish

Pretreatment: The industrialization challenges faced by farmers and producers in the crayfish industry, such as seasonal production and sales, have limited their development. In recent years, the crayfish processing industry has experienced rapid growth to address these issues. As an aquatic crustacean that grows in swamps and wetlands, thorough cleaning before processing is essential for ensuring food safety. The food industry has seen a trend towards the use of non-thermal techniques in the disinfection of food products due to their minimal impact on the texture, appearance, aroma, and nutritional composition of food products. Non-thermal disinfection techniques encompass high-pressure treatment, low-temperature plasma, ultrasound technology, ultraviolet radiation application, intense pulsed light utilization, and chemical disinfectants. Ultra-pressure treatment causes partial protein denaturation and muscle tissue tightening, resulting in increased hardness and reduced springiness. Treatment at 200 MPa for 5 min is optimal for peeling crayfish and preserving meat quality [31]. It has been reported that moderate ultra-high-pressure treatments (less than 300 MPa) could be applied to modify the protein structure and water distribution of crayfish muscle [32]. However, these critical technologies and processes for the production of the above products are immature, which to some extent limits the in-depth development of the crayfish industry. Ultrasound followed by ozone-water cleaning showed a better effect on the reduction of the total viable count (TVC) of crayfish than ozone-water cleaning followed by ultrasound cleaning and simultaneous ozone-water and ultrasound cleaning. The samples subjected to ozone-water cleaning treatment showed myofibril separation in tail meat and an increase in the pH, the myofibril fragment index, and thiobarbituric acid reactive substance (TBARS) content, but no significant effect on physical and sensory qualities was found [33]. Recently, a decontamination technology that combines ultrasound and plasma-activated water was proposed and found to be more useful in microbial decontamination compared to the natural microbiota of crayfish, effectively inhibited microbial growth, and suppressed the oxidation of proteins and lipids during storage, resulting in a longer shelf life, and also effectively delayed the degradation of the textural and sensory properties of crayfish [34].

Processing: The current processed product categories mainly include frozen boiled crayfish flesh, quick frozen cooked crayfish tails, quick frozen cooked whole crayfish, etc. [35]. Research into crayfish processing methods has demonstrated that different techniques yield varying effects on crayfish quality(Table 4). Generally, thermal treatment is a crucial step in the production of crayfish products. Elevated temperatures can effectively eliminate most surface and internal microorganisms in crayfish, deactivate enzymes, and reduce subsequent sterilization time, thereby enhancing product safety. Additionally, it can facilitate moderate nutrient hydroxylation, promoting improved human digestion and absorption. Furthermore, heating results in bright red coloration, an elastic and dense texture, as well as a distinctive flavor in crayfish. A study of the effect of cooking temperature and duration on the physicochemical, textural, structural, and microbiological characteristics of fresh crayfish showed that crayfish cooked at 93–95 °C for 3–5 min had the highest hardness at around 330–373 g. Higher temperatures also significantly reduced the total viable count [35]. However, as the heating time increased, water loss and damage to the endomuscular membrane caused the muscle fiber microstructure to change from a compact to a loose state. The effect of different cooking techniques such as steaming (100 °C), boiling (100 °C), frying (160 °C), and high-pressure steaming (121 °C) on the quality of crayfish was compared. The astaxanthin content in crayfish increased significantly after cooking, and the content in the steamed crayfish (81.43%) was significantly higher than other cooking methods. The results indicated that both steaming and boiling resulted in superior taste and texture, while steaming also proved to be more effective in preserving the edible quality of crayfish meat due to its lower content of volatile compounds compared to frying, suggesting that steaming could be better for crayfish meat to maintain the edible quality [36]. Microwave heating caused nutrient loss within acceptable ranges, but significantly decreased the fresh flavor and taste of crayfish following a linear trend. The effects of microwave and boiling on the quality of crayfish tail were studied using the visualization method, providing a new strategy for food cooking evaluation, and the loss of moisture during microwave heating of crayfish tails was much lower than that of whole crayfish when the tails were heated selectively and cooked rapidly [37].

*Preservation:* Crayfish quality inevitably changes during storage. Enzymes such as trypsin-like proteases, cathepsin B, polyphenol oxidase, prophenol oxidase, and ATPase play important roles in proteolysis, melanin formation, and adensine triphosphate degradation, leading to muscle softening, melanosis, and loss of umami [38]. More importantly, microbial activity is the main cause of off-flavors in crayfish during cryopreservation. Storage technology is key to processing and circulation. Commonly used storage technologies include freezing, ultra-high-pressure treatment, the addition of preservatives, edible coating, and modified atmosphere packaging (Table 5). These storage methods focus on delaying the decomposition and deterioration of crayfish internal components, microbial growth and propagation, and reducing enzyme activity. At faster freezing rates, the formation of small intracellular ice crystals with minimal mechanical damage to structural properties would preserve cell membrane integrity to maintain supercooling status, contributing to better biochemical properties [39]. The freezing temperature (liquid nitrogen, −80, −30, and −18 °C) and storage time (1, 4, 12, and 24 weeks) on the properties of red swamp crayfish were studied [40]. The results showed that the freezing/storage temperature difference and freezing rate influenced the final quality of crayfish products, and freezing at −30 °C could be considered a suitable processing method for crayfish. Biochemical characterization results showed that liquid nitrogen freezing was helpful in inhibiting quality degradation, such as reducing TVC, TVB-N, and TBA, decreasing α-glucosidase and β-glucosidase enzyme activities, and delaying protein denaturation.

Edible coatings can effectively inhibit the reproduction of spoilage bacteria and enzyme activity during storage and delay the oxidation and decomposition of fat, thus maintaining product quality for a longer period. The application of an edible coating during crayfish storage can effectively inhibit the breeding of spoilage bacteria and enzyme activity and delay the oxidation and decomposition of fat, thus maintaining product quality for a longer period [41]. The packaging of shelled crayfish with an edible coating containing red pitaya peel extract (RPPE) and ε-polylysine (ε-PL) retarded the quality deterioration of shelled crayfish during storage, and the coating combined with 2.0% RPPE was found to be preferred [42].

Modified Atmosphere Packaging (MAP) is an innovative packaging technology utilized for the preservation of meat and meat products, effectively extending their shelf life without the need for preservatives [43]. MAP involves carefully adjusting the composition of gases surrounding the food within a package, with oxygen to prevent anaerobic growth and maintain color, carbon dioxide to inhibit microbes, yeast, and mold, and nitrogen to prevent package collapse. Compared to traditional aerobic packaging, MAP significantly reduces microbial growth while ensuring the market quality and safety of meat products. Cremades revealed that by packaging cooked crayfish tails in a modified atmosphere consisting of 60% N_2_ and 40% CO_2_, the deterioration of quality was delayed from 6 days to 11–12 days [44]. The safety of MAP might become a concern when meat preservation was subjected to elevated storage temperatures, as it may lead to the proliferation of anaerobic spoilage and pathogenic microbes [43]. In this situation, smart label sensors with predictive models to indicate the quality of meat packed in a modified atmosphere are a potential technology to monitor the quality and safety of ready-to-eat foods like crayfish.

A variety of preservation methods are currently available, and utilizing biological preservatives instead of chemical ones can circumvent the impact of high-temperature sterilization on product quality. However, there remains a challenge in achieving sufficient sterilization and bacteriostatic effects when transitioning from single cryogenic storage technology to a combination of cryogenic and other preservation technologies. A study of the effects of lactic acid on the growth and survival of *Listeria monocytogenes* in crayfish tail meat stored under refrigerated and various gaseous environments revealed that the combination of lactic acid and a modified atmosphere has significant potential to inhibit *L. monocytogenes* growth [45]. Furthermore, employing low-concentration complex organic acids alongside autoclave treatments has shown promise in enhancing the quality characteristics of crayfish meat, including texture, color, and sensory attributes, thereby presenting a novel approach for developing ambient storage crayfish products [46]. Additionally, microwave treatment in combination with sodium lactate and the antimicrobial peptide nisin has shown significant efficacy in inhibiting the spoilage of crayfish tails, as evidenced by the reduced total viable count after 3 days of storage at room temperature, which meets the transit time requirements of most logistics companies in China [47].

A key finding of this review is that although over 90% of international trade is based on processed products, live fish/shellfish are particularly appreciated in Asia and other niche markets where aquariums and tanks displaying live fish are increasingly common in seafood restaurants, supermarkets, and retail outlets. Particularly, people prefer to consume fresh crayfish, preferably when they can see it alive before processing; in this case, keeping alive is more important than preservation. The stress response, caused by exposure to adverse environmental conditions or physical handling, is an adaptive mechanism to cope with stressors in order to maintain a homeostatic state. It involves a series of sequential events (responses), beginning with an initial neuroendocrine response that results in changes in appearance and quality [48,49]. It has been studied in many different vertebrate and invertebrate species. For example, during the long-time transportation of white leg shrimp (*Penaeus vannamei*), the pH of the water and the content of total ammonia nitrogen and non-ionized ammonia were elevated. Shrimp muscle water holding capacity, hardness, and shear were also reduced with intensive myofibrillar protein degradation [50]. Packing in pre-cooled sawdust or wood shavings is used to minimize stress during transport. Black tiger shrimp (*Penaeus monodon*) and freshwater shrimp (*Macrobrachium rosenbergii*) can be packed in plastic bags containing water and oxygen, and softshell blue crabs are shipped at 4 °C in moist marsh grass or newspapers [51]. At present, it mainly focuses on the development and application of preservation technology for crayfish, and researchers need to focus on crayfish habits and study how to keep crayfish alive during long-term logistics and shelf life.

**Table 4:** Various processing methods on the quality of the crayfish.

Processing Methods	Effects	Reference
Steaming	Excellent taste; good hardness and chewiness; low cooking loss rate; low fat oxidation degree.	[36]
Frying	More cooking loss; high fat oxidation degree; more volatile compounds.	[36]
High-pressure steam	More cooking loss; high astaxanthin content; good smell; decrease in springiness.	[36]
Microwave	More cooking loss; high cooking uniformity.	[37]
Sous vide cooking	Less cooking loss; good texture; low TBA value.	[52]

**Table 5:** Various preservation technologies on the quality of the crayfish.

Technologies	Conditions	Effects	Reference
Freezing treatment	Storage at freezing temperature with liquid nitrogen (−80, −30, and −18 °C) for 1, 4, 12, and 24 weeks	The Ca^2+^-ATPase activity, the salt soluble protein content, and the total and reactive sulphhydryl content of the myofibrillar protein extracted from crayfish were significantly decreased (*p* < 0.05), and the expressible moisture was significantly increased (*p* < 0.05). The recommended shelf life for crayfish is 1 month.	[40]
Edible coating	A solution of chitosan containing propolis extract emulsions	Compared to the control group, the shelf life of the crayfish was increased by 7 days.	[41]
	Gelatin incorporated with red pitaya peel methanol extract	The values for total volatile basic nitrogen (TVB-N), K maintenance, and free amino acids (FAAs) decreased significantly (*p* < 0.05).	[42]
MAP	60% N_2_ and 40% CO_2_	Inhibited the growth of psychrophilic bacterial, hydrogen sulfide-producing bacteria, and Enterobacteriaceae, reduced the content of TVB-N, and inhibited lipid peroxidation.	[44]
Ultrasound assisted	Alginate oligosaccharide (1%, *w*/*v*) with ultrasound-assisted (40 W, 3 min) soaking	Increased the water retention, α-helix and β-fold content of cooked crayfish after five freeze–thaw cycles, and contributed to the structural stability of myofibrillar protein.	[53]
	Ultrasound-assisted chitosan nano-composite water retaining agent	TVB-N, the content of myofibrillar protein, and the Ca^2+^-ATPase activity of the muscle protein were significantly delayed, thus preserving the integrity of the tissue structure.	[54]
Combination	Complex organic acids, high-temperature sterilization	Improved meat quality after sterilization, such as texture, color, and sensory characteristics.	[46]
	Microwave, sodium lactate, and the antimicrobial peptide nisin	Significant inhibition of spoilage as measured by total viable count (4.15 log CFU/g) after 3 days storage at room temperature.	[47]

## 5. Utilization of By-Products

**Figure 3:**
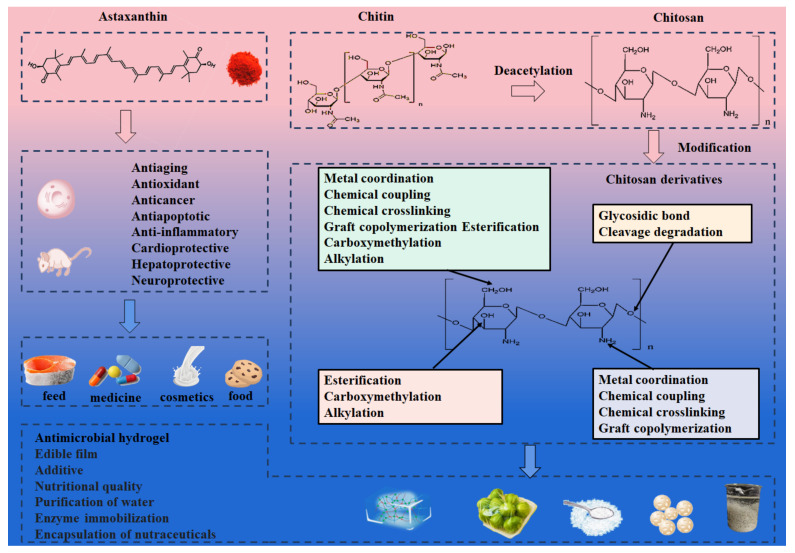
Nutritional/bioactive values and applications of the typical by-products (astaxanthin and chitosan).

Traditional processing results in approximately 80% of crayfish shells being wasted annually, generating around 100,000 tons, and improper disposal contributes to environmental pollution, while appropriate disposal can be costly [55]. Crayfish shells have a unique composition made up of protein (20–30%), calcium carbonate (30–40%), and chitin (20–30%). Minor components such as lipids, astaxanthin, and other minerals have also been identified. Hence, the retrieval and isolation of bioactive compounds from shell waste are imperative for environmental conservation and the efficient management of waste [56]. The exploitation of abundant crayfish shell resources has garnered growing interest among researchers, as shown in Figure 3.

### 5.1. Extraction and Application of Astaxanthin

Astaxanthin (3, 3′-dihydroxy-β, β-carotene-4, 4′-dione) is a lipophilic carotenoid categorized under the xanthophyll group [57], and due to its numerous favorable properties and health benefits, is widely utilized in the food, cosmetics, and pharmaceutical industries(Table 6), as well as other related fields as a more potent antioxidant compared to ascorbic acid, tocopherol, and β-carotene. Various extraction methods (Table 7) such as chemical, alkali, ultrasound, oil, and supercritical fluid extraction have been employed for improving its production [24,58]. These techniques are based on the principle of “similar compatibility” to dissolve the target product in the solvent. Although simple to perform, they often result in low yields due to astaxanthin’s strong antioxidant properties, which make it susceptible to degradation during the extraction process. The highest yield of astaxanthin extracted with isopropanol/n-hexane (50:50 vol%) was 43.9 µg/g wet waste, exceeding that extracted with pure acetone (40.6 µg/g wet waste) and isopropanol (40.8 µg/g wet waste) [59]. Compared to conventional organic solvents, ionic liquids for extraction have shown great promise in improving the selectivity and extraction yields of bioactive compounds in samples, as well as reducing environmental impact. Under optimized conditions, the ionic liquid almost doubled the extraction of astaxanthin from mussel waste compared to the conventional method, but it still required different organic solvents for washing and elution [60]. A high-pressure process for the extraction of astaxanthin from shrimp shells increased the yield from 29 µg/g (dry weight) to 60 µg/g in comparison with chemical extraction [61]. As can be seen from Table 7, it has demonstrated that chemical processing leads to challenges for disposal and safety; physical and fermentation methods have been developed for processing shells, but they are time consuming and require specialized equipment. In contrast, enzymatic treatment offered excellent adaptability, scalability, environmental friendliness, and energy efficiency. Strategies for converting shell biomass into usable products without wasting shell could provide both economic and environmental value. A cost-effective enzymatic process utilizing proteases and chitinase has been developed for the recovery of shrimp shell waste. For example, one gram of waste shell yields 101.3 μg of astaxanthin using ethyl acetate after enzymatic hydrolysis of protein and chitin, while maintaining natural biological activities despite a relatively longer incubation time [62]. At a neutral protease concentration of 20 u/g, an enzymolysis temperature of 50 °C and an enzymolysis time of 1 h, the yield of astaxanthin reached 134.20 μg/g, which was 3.7 times higher than that of the control group of 36.03 μg/g. This indicates that the use of neutral protease for enzymatic hydrolysis of shrimp shells could significantly improve the extraction of astaxanthin [63].

Furthermore, comprehensive research is required to extract astaxanthin from crayfish using diverse microorganisms that offer significant benefits to human health as well as the environment and are strongly recommended. A more environmentally friendly extraction of astaxanthin using two bacterial and fungal probiotics (*Bifidobacterium lactis, Lactobacillus lactis, Candida utilis,* and *Saccharomyces cerevisiae*, respectively) instead of chemical processes resulted in the highest concentration of extracted astaxanthin using *S. cerevisiae* (45.69 µg/g), and the inhibition of hemolysis suggests that crayfish with astaxanthin treated with *S. cerevisiae* might have more anti-inflammatory activity [64].

**Table 6:** Typical utilization of astaxanthin.

Application	Effect	Reference
Colorant	Astaxanthin supplementation increased astaxanthin levels in the Chinese mitten crab (Eriocheir sinensis).	[65]
	Used in aquaculture to pigment the flesh of salmon, trout, and shrimp (these animals do not synthesize astaxanthin de novo), influencing consumer preferences around the world.	[66]
Feed	Astaxanthin supplementation in aquaculture diets improved the growth performance and survival of Asian perch.	[67]
	The addition of astaxanthin to the diet of Atlantic cod (*Gadus morhua L.*) increased fertilization, improved the survival of the larvae, and reduced the mortality of the embryos.	[68]
	The addition of astaxanthin-rich skullcap powder to the diet of coral trout improved their digestive enzyme activity, antioxidant capacity, and immunity.	[69]
Food	Cookies with 15% astaxanthin had significantly lower free glucose release.	[70]
	Astaxanthin inhibits the oxidation of free docosahexaenoic acid through the binding of oxygen radicals for food preservation.	[71]
	The encapsulated astaxanthin-containing lipid showed higher bioaccessibility and antioxidant activity than the non-encapsulated sample when it was prepared by spray-drying using a variety of wall materials.	[72]
Pharmaceuticals	Astaxanthin reduced the expression of CYP2E1, increased the sensitivity of cells to insulin, and inhibited damage to the liver.	[73]
	Astaxanthin inhibited ischaemia-induced cell death in the retina through its antioxidant activity.	[74]
	Astaxanthin supplementation in rats after surgery reduced the expression of NF-κB and TNF-α, resulting in reduced brain edema and neurologic dysfunction.	[75,76]

**Table 7:** Summary of methods for extracting astaxanthin.

Method	Advantages/Limitations	Conditions	Yield	Reference
Ultrasonic-assisted	Convenient and efficient; short duration; high extraction rate.; may lead to some degradation of astaxanthin.	Ethanol, ultrasonic frequency of 40 kHz over 20 min	239.96 µg/g	[77]
		0.50 mol/L [C_3_NH_2_MIM][Br] in ethanol as the extraction solvent, ultrasonic power of 75 W for 60 min	92.7 µg/g	[60]
High-pressure	Higher astaxanthin yield; higher quality; shorter extraction time.	210 MPa for 10 min at room temperature	59.97 µg/g	[61]
Chemical extraction	Easy operation; simple equipment.Large solvent consumption; may cause environmental pollution.	A mixture of acetone and methanol (7:3, *v*/*v*), centrifuged for 10 min at 10,000 g	29.44 µg/g	[61]
		Ethanol, 50 °C, 20 min	43.7 µg/g	[77]
		Ethyl acetate, 30 °C, 2 h, 150 rpm, after enzymatic hydrolysis	101.3 µg/g	[62]
Oil extraction	Safety; less pollution; delay the oxidation and degradation of astaxanthin; oil is not easy to remove after extraction; high temperature may affect the stability.	Sunflower oil, 70 °C, 150 min	27.56 µg/g	[78]
		Flaxseed oil, 60 °C, 60 min	30.2 µg/g	[79]
Enzyme extraction	Mild reaction conditions; reduced energy usage; eco-friendly; costlier; long enzymolysis time.	Neutral protease, 50 °C, 60 min	134.20 µg/g	[63]
Supercritical CO_2_ extraction	Environmentally friendly; shorter extraction time; no organic solvent residue; high maintenance cost of the equipment.	CO_2_ + 10 wt % ethanol, 60 °C, 224 bar, 25 wt% moisture	177.58 µg/g	[80]
Biological extraction	Eco-friendly; long time.	*Saccharomyces cerevisiae*	45.69 µg/g	[64]

### 5.2. Extraction and Application of Chitin and Its Derivatives

Chitin is a polysaccharide consisting of β-1,4-linked N-acetyl-D-glucosamine. It is the second most common renewable resource in nature, second only to lignocellulose, and is found in crustacean shells, insect cuticles, and the cell walls of fungi [81,82]. Demineralization, deproteinization, and decolorization are typically key steps in the extraction process [83]. A variety of methods, including physical, chemical, enzymatic, hydrolytic, and biological techniques, can be used to extract chitin from crayfish shells (Table 8). The pretreatment of crayfish shell waste with instant catapult steam explosion results in lower crystallinity and increased surface area, significantly improving chitin extraction efficiency [77]. The modification of traditional chemical methods by introducing sodium hypochlorite before demineralization and deproteinization can reduce the total time required for chitin extraction from 1 day to 1 h [84]. Although chemical processing is commonly used in commercial industries due to its short processing time and high efficiency, it generates large amounts of toxic waste without effective treatment that are harmful to the environment. Additionally, alkali treatment may impact the quality of chitin. Therefore, biological extraction has gained attention for its environmental friendliness and cost-effectiveness. A combination of enzymatic hydrolysis and fermentation with proteinase and *Bacillus coagulans* can achieve high rates of deproteinization (91%) and demineralization (94%) after 48 h of fermentation for chitin extraction from crayfish shell waste [85].

Chitosan, a modified natural carbohydrate polymer derived from chitin deacetylation, is influenced in its degree of deacetylation and physicochemical properties by the components of the raw materials and the method of preparation. Shrimp and crayfish shells are found to be the optimal source for chitosan extraction compared to fish and crabs when using 40% NaOH for chitin deacetylation and measuring physicochemical properties [86]. Chitosan extracted from crayfish exoskeleton waste yielded a degree of 87% deacetylation [87]. Due to its multiple physiological functions including biodegradability, biocompatibility, non-toxicity, bacteriostasis, and lipid-lowering and immunomodulatory properties [88], chitosan has found extensive applications in various fields, including food additives, environmental protection, cosmetics, antimicrobial agents, medical fibers, medical dressings, and biomedical research, as has been documented in numerous literary works (Table 9).

As a conclusion for the utilization of crayfish by-products, a variety of extraction methods for astaxanthin and chitin as well as its derivative chitosan exhibit distinct advantages and limitations. Chemical extraction offered ease of operation but came with high energy consumption and the potential for introducing harmful pollutants to the environment. Ultrasound-assisted extraction is a convenient and efficient method due to its short duration and high extraction rate, although it may lead to some degradation of astaxanthin. Supercritical CO_2_ extraction stands out as an environmentally friendly and safe approach for extraction and separation; nevertheless, the high maintenance cost of the equipment hinders industrial production. Microbial fermentation and enzymolysis provided mild reaction conditions, reduced energy usage, and eco-friendly benefits; however, bio-enzymatic preparations are costlier and enzymolysis requires extended processing time. It is imperative to explore ways to enhance extraction rates in conjunction with other methods given that existing techniques often entail significant costs or time requirements despite efforts to recycle chemical substances from crayfish shells, resulting in limited recycling of crayfish-shell waste through these means. Consequently, there is a critical need for the development of new, economical, straightforward technologies capable of efficiently recycling crayfish shells.

**Table 8:** Summary of the methods for extracting chitin.

Method	Advantages/Limitations	Conditions	Yield	Reference
Fermentation and hydrolysis	Eco-friendly; high deproteinization rate; high demineralization rate; high recovery rate; long duration.	Hydrolysis with *Bacillus coagulans* LA204 and proteinase K. A simultaneously fermented at 50 °C for 48 h.	94%	[85]
Endogenous enzyme autolysis and fermentation	Environmentally friendly; safety; high deproteinization rate; many influencing factors.	Autolyzed with a natural pH at 50 °C for 4 h, then fermented by *Bacillus licheniformis* at 60 °C for 10 h, and the residue was demineralized with 10% citric acid to obtain chitin.	-	[89]
Biological extraction	Environmentally friendly; low cost; low degree of depolymerization; long time; incomplete removal; many influencing factors.	Autolysis at pH = 2.	14.7%	[90]
		Fermentation with *Bacillus licheniformis* 21886 and *Gluconobacter oxydans* DSM-2003.	-	[91]
		*Lactobacillus delbrueckii* performed demineralization, and *Bifidobacterium lactis* led the deproteinization.	-	[92]
		*Bacillus bacteria* for fermentation.	-	[93]
Chemical extraction	Easy operation; low cost; long extraction time; high resource consumption; environmentally unfriendly.	Using hydrochloric acid in demineralization and sodium hydroxide in deproteinization.	20%	[94]

**Table 9:** Some examples for the utilization of chitosan.

Application	Effects	Reference
Biomedicine	In mouse tail amputation, femoral artery hemorrhage, and liver incision models, an injectable hydrogel of quaternary ammonium chitosan and tannic acid was able to rapidly stop bleeding.	[95]
	Chitosan hastened wound healing through interactions between its amino groups and platelets.	[96]
	Water-soluble chitosan mouthwash showed lower toxicity and higher antimicrobial activity than commercial mouthwashes with or without alcohol. *Streptococcus mutans* and *Lactobacillus brevis* were significantly inhibited by chitosan antibacterial activity.	[97]
Environment	After treatment with chitosan and subsequent filtration, the final turbidity of the seawater was significantly reduced.	[98]
	Hierarchically structured composite scaffolds synthesized by freeze casting hydroxyapatite powder followed by chitosan crosslinking showed great ability to remove heavy metal ions from wastewater.	[99]
Food	Treatment with chitosan nanocomposite water retention agent significantly reduced thawing loss, water activity, and TVB-N content of crayfish.	[54]
	Due to chitosan’s antimicrobial and antioxidant properties, it has been used as a bio-preservative in the meat industry.	[100]
	Chitosan has been used as a natural flocculant for beer clarification. It reduces the zeta potential, viscosity, and phenolic content of beer.	[101]
Cosmetics	Chitosan, added to toothpastes and mouthwashes to prevent biofilm formation due to the presence of *mutans streptococci* in the mouth, has been shown to reduce *mutans streptococci* colonies in early childhood caries.	[102]
	Chitosan nanoparticles could be used as a cosmetic and dermal drug delivery system, with the ability to deliver active ingredients and cosmetic components through the skin.	[103]

## 6. Safety Evaluation

The factors affecting the safety of crayfish products can be broadly categorized into two types: microbial and chemical pollutants. Microorganisms include pathogenic bacteria, parasites, and viruses, whereas chemical pollutants include heavy metals, pesticide residues, and veterinary drug residues. Crayfish are similar to many other aquatic organisms in terms of pesticide residues and microbials-related safety issues, so instead of discussing them here, we focus on heavy metal and drug enrichment due to crayfish growth habits. In addition, another risk of consuming crayfish are allergens.

Heavy metals are a group of metals with relative densities greater than 4.0. Elements like lead (Pb), cadmium (Cd), and chrome (Cr) and metallic elements like arsenic (As) may increase the risk of cardiovascular disease, teratogenicity, skin disorders, and cancer [104,105,106]. Mercury (Hg) damages the central nervous system, while inorganic Hg compounds damage the kidneys [107]. Crayfish have strong accumulation characteristics for heavy metals, and the heavy metal content in their bodies may be several times that of the surrounding environment. Therefore, the problem of heavy metal residues in crayfish has been a concern. The exposure of crayfish to Cd causes histological alterations in the intestines and alters the richness, diversity, and composition of the intestinal microbiota [108]. The concentrations of Cr, As, Pb, Cd, and Hg were recorded in the abdominal muscle, gonads, and hepatopancreas. The hepatopancreas is the primary organ for Cd, As, and Pb deposition, the abdominal muscle is the ideal organ for Cr and Hg deposition, and the gonads are the primary organs for As deposition [107]. The concentrations of copper (Cu), Cr, Cd, zinc (Zn), and Pb were measured in the water and sediment, and in the muscles and exoskeletons of crayfish. The bioaccumulation of heavy metals in crayfish is within standard guidelines, except in highly polluted drains. It is recommended that crayfish in drainage and contaminated waterways should not be consumed by humans, as muscles and exoskeletons specifically accumulate Hg and Ni, respectively; meanwhile, Cd, Zn, Cu, Pb, and Cr primarily accumulate in the hepatopancreas [109]. There were significant differences in the distribution of heavy metals in wild and farmed crayfish. In general, the average concentration of heavy metals is higher in the wild crayfish than in the cultured crayfish. In both farmed and wild crayfish, hepatopancreas was the most concentrated tissue, followed by gills, exoskeleton, and abdominal muscle [110]. The accumulation sequence of the elements in the different tissues was gill > foot > muscle, with most of the elements accumulating in the gill tissues, whereas Hg was concentrated in the abdominal muscle and Zn in the foot tissues. It is worth noting that the levels of toxic elements were lower in the abdominal muscle than in the exoskeleton and hepatopancreas, which are not recommended for consumption [111].

In order to obtain better quality crayfish, some pharmaceuticals may be used in the breeding process. Pyrethroid pesticides are used for pest control in both agriculture and aquaculture. The study showed that deltamethrin induces DNA damage, immunotoxicity, and neurotoxicity in crayfish through the excessive generation of reactive oxygen species (ROS) [112]. Deltamethrin is acutely toxic to crayfish hemolymph, gill, muscle, and liver, and the study showed LC50s for 24, 48, and 96 h of 0.156, 0.099, and 0.056 μg/L, respectively [113]. Diclofenac (DCF) is an anti-inflammatory drug widely used worldwide for veterinary and medical purposes. It can induce the differential expression of immune- and redox-related genes in crayfish, and regulate the processes of molting, amino sugar metabolism, proteolysis, and intracellular protein transport in crayfish. In addition, DCF can alter the relative abundance of microbial families in the gut, leading to the disruption of the gut microbiota, which may further contribute to intestinal metabolic dysfunction in crayfish [114].

Additionally, serious allergic reactions can occur in some people who consume crayfish. Common symptoms include skin symptoms, gastrointestinal reactions, and systemic symptoms such as rashes, vomiting, respiratory distress, and shock in severe cases, which can be life threatening. Tropomyosin, arginine kinase, triose-phosphate isomerase, and hemocyanin subunits are the main identified and characterized allergens of crayfish [115,116]. Tropomyosin, a thermally stable myofibrillar protein consisting of two subunits with molecular masses of 36–38 kDa, is the major allergen in crayfish, which has been purified and immunologically characterized. Now, research on other allergens and cross-reactivity is being strengthened gradually. Chen et al. identified crayfish arginine kinase as an allergen, cloned the protein for B-cell epitope prediction, and evaluated physicochemical, processing stability, and immunological properties [117]. The sarcoplasmic calcium-binding protein with a molecular mass of 22 kDa was also confirmed as a novel red crayfish allergen by mass spectrometry determination of its IgE-binding activity. Physicochemical characterization showed it to be a highly stable allergen, and it was recently reported to be cross-reactive with triosephosphate isomerase [118]. Myosin light chain myosin and light chain isoform 1 were reported to be novel allergens in crayfish by Zhang et al. [119] and Yang et al. [120], respectively. Crayfish light chain isoform 1 showed a high degree of primary and secondary structural identity with myosin light chain, its epitopes were located in the structurally conserved regions, and its cross-reactivity between related species was demonstrated by immunological assays.

With the increasing prevalence of crayfish allergy, scientifically sound processing methods have become an important option available for preventing allergic subjects from anaphylactic reactions; for example, light chain isoform 1 was stable at 30 to 100 °C and under highly acidic and alkaline conditions, and retained its IgE-binding activity at different temperatures and pH values. The sarcoplasm calcium-binding protein is a stable polymorphic allergen in crayfish, and all its isotypes and subunits are allergenic. The common desensitization processing technologies are ultra-high pressure treatment [121], high intensity ultrasound [122], radiation reduction [123], enzymatic treatment [124], etc. Food-sourced chemical reagents or food ingredients could be a novel way applied to specifically eliminate allergic reactions, with the advantages of fewer adverse effects, high safety, and low cost [125]. Recently, the immunoregulatory properties of natural bioactive compounds, such as polyphenols, polysaccharides, and oligosaccharides, have been extensively studied. This suggests their potential in the prevention and treatment of human diseases. However, the utilization of natural products for eradicating crayfish allergens has been infrequently documented. Chlorogenic acid, as one of the most abundant acids within the realm of phenolic acid compounds, naturally occurring in extracts of green coffee and tea, can address crayfish allergies by reducing the sensitizing properties of tropomyosin through covalent or noncovalent binding, altering its secondary structure and masking the linear epitope of tropomyosin [126].

## 7. The Challenge and Opportunity from Crayfish

As a well-known species, crayfish has been exploited and utilized by humans in many areas of the world. The availability and nutritional value have made it as a non-negligible source of food and economic development in many regions. It has been reported that Louisiana’s crayfish industry was worth more than $200 million in 2016, and the high profitability of the crayfish industry has led to attempts to replicate its aquaculture production in other regions, for example, in Kenya and Spain [1]. However, opportunities and challenges often coexist. Among crustaceans, crayfish in particular have been introduced on a large scale and are considered to be a major threat to the functioning of freshwater ecosystems. The first reports of the introduction of crayfish outside their natural range date back to the year 1746 [127]. Invasive crayfish can significantly disrupt the ecological functioning of a freshwater system due to their ability to occupy multiple trophic levels due to their omnivorous diet [128]. Invasive crayfish impact on native crayfish mainly through competition, predation, disease introduction, and reproductive effects [129]. Various methods have already been applied and tested to reduce or eradicate the negative impacts of crayfish invasions. These include traditional techniques such as physical controls, barriers and drainage interventions, biological control, and biocidal control, as well as emerging techniques such as sex attractants, the silencing of key hormones through RNA interference (RNAi), and oral delivery [130]. The introduction of non-native species as an economic species requires extreme caution and rigorous verification. In the process of circulation and consumption, crayfish should be tested to control species invasion. New and emerging techniques for the detection and control of invasive crayfish and the protection of endangered native species are now highly desirable, and several are being evaluated. For example, eDNA has been developed over the last few years to detect both non-native and native crayfish, making it possible to detect target species at low levels and where they cannot be directly observed [130].

## 8. Conclusions

The crayfish, abundant in proteins, minerals, trace elements, and a variety of vitamins, is an aquatic delicacy with exceptional nutritional value. The future will see continued research and development in the nutritional composition, functional components, and bioactive properties of crayfish. In addition, the advancements in crayfish deep processing techniques are demanded to create products such as fried/dried crayfish meat and crayfish-sourced sauce, which would involve flavor retention problems during processing and fermentation manufacturing. Moreover, various preservation technologies, especially the technology of keeping alive, should be developed and combined to overcome seasonal or regional limitations and develop safe and efficient methods for preserving crayfish. These innovations will expand the utilization of crayfish and open up new opportunities for the growth of the industry.

Driven by increasing market demand, the crayfish industry is expected to maintain a medium-to-high growth rate. In this context, the efficient utilization of such a scale of waste or by-products will present itself as both an opportunity and a challenge. Intensive processing will also progress steadily with a focus on efficiently utilizing by-product resources from crayfish to produce high-value items like enzyme preparations and chitosan microcapsules that can find applications in biomedicine, cosmetics, food, fertilizers, and environmental protection materials. Furthermore, efforts will be made to upgrade extraction technology to explore new environmentally friendly methods for effectively utilizing by-product resources from crayfish, as only a single component of crayfish shell can be recovered through traditional chemical processes, and the ideal by-products’ comprehensive utilization should be biorefining combing with other eco-technologies, which are proposed to separate them into different fractions that can be converted into valuable products.

## Figures and Tables

**Table 1:** The basic nutrients in crayfish (%) analyzed in various references.

Component	% Wet Basis	% Wet Basis	% Wet Basis	% Wet Basis	% Dry Basis
	Wild	Farmed	Farmed	Wild	Wild
	[4]	[5]	[6]	[7]	[8]
Moisture	82.15 ± 0.03	80.81 ± 0.03	76.97 ± 0.21	-	75.75 ± 2.30
Crude protein	15.22 ± 0.07	18.23 ± 0.20	19.48 ± 0.23	13.88 ± 1.48	73.25 ± 4.10
Crude fat	1.29 ± 0.03	1.24 ± 0.03	0.89 ± 0.01	1.76 ± 0.08	7.56 ± 0.60
Crude ash	1.18 ± 0.02	1.23 ± 0.02	1.41 ± 0.01	1.52 ± 0.14	9.30 ± 3.90
Carbohydrates	0.16 ± 0.21	-	-	-	1.86 ± 1.10

**Table 2:** Amino acids’ patterns in crayfish (%) in different literatures.

Amino Acids	% (Wet Weight)	% (Dry Weight)	% (Dry weight)	% (Dry Weight)	mg/100 g
	Wild	Farmed	Farmed	Wild	Farmed
	[10]	[5]	[6]	[7]	[13]
Aspartic acid	1.58 ± 0.04	1.75 ± 0.01	2.045 ± 0.021	9.48 ± 0.924	26.72 ± 1.11
Threonine	0.71 ± 0.02	0.64 ± 0.02	0.735 ± 0.004	1.494 ± 0.231	192.88 ± 11.77
Serine	0.57 ± 0.01	0.65 ± 0.01	0.745 ± 0.010	2.891 ± 0.389	360.25 ± 18.5
Glutamic acid	2.66 ± 0.06	3.27 ± 0.03	3.107 ± 0.006	11.207 ± 1.120	289.20 ± 73.89
Proline	0.35 ± 0.01	0.31 ± 0.04	0.594 ± 0.054	4.006 ± 0.223	-
Glycine	0.68 ± 0.03	0.82 ± 0.03	0.876 ± 0.016	5.815 ± 0.413	1344.55 ± 101.06
Alanine	0.80 ± 0.01	1.03 ± 0.01	1.076 ± 0.023	5.59 ± 0.521	954.62 ± 191.31
Cystine	0.11 ± 0.00	0.18 ± 0.02	0.216 ± 0.003	0.339 ± 0.188	-
Valine	0.66 ± 0.01	0.73 ± 0.02	0.876 ± 0.015	6.707 ± 0.620	204.65 ± 17.65
Methionine	0.27 ± 0.00	0.39 ± 0.02	0.416 ± 0.024	5.928 ± 0.296	201.09 ± 3.64
Isoleucine	0.76 ± 0.01	0.72 ± 0.01	0.842 ± 0.020	6.699 ± 0.178	120.95 ± 5.19
Leucine	1.36 ± 0.02	1.36 ± 0.03	1.492 ± 0.027	9.831 ± 6.057	224.86 ± 37.04
Tyrosine	0.40 ± 0.00	0.57 ± 0.02	0.696 ± 0.019	2.297 ± 0.110	238.04 ± 64.22
Phenylalanine	0.63 ± 0.01	0.69 ± 0.01	0.785 ± 0.016	4.104 ± 0.279	103.65 ± 34.02
Histidine	0.42 ± 0.01	0.49 ± 0.02	0.451 ± 0.011	3.0944 ± 0.256	42.96 ± 7.66
Lysine	1.40 ± 0.04	1.42 ± 0.01	1.580 ± 0.030	6.462 ± 0.627	-
Arginine	1.52 ± 0.05	2.03 ± 0.02	2.181 ± 0.026	11.384 ± 1.48	6529.96 ± 500.55

**Table 3:** Fatty acid content in different freshwater crayfish samples.

Fatty Acid	‰ Fresh Sample	% Total Fatty Acids	% Total Fatty Acids	% Dry Weight
	[5]	[10]	[6]	[8]
C12:0	0.01 ± 0.00	0.13 ± 0.00	-	0.09
C14:0	0.11 ± 0.00	0.47 ± 0.09	0.49 ± 0.02	1.40
C15:0	0.13 ± 0.00	1.25 ± 0.19	0.46 ± 0.01	1.30
C16:0	2.70 ± 0.00	15.74 ± 0.70	16.45 ± 0.29	26.63
C17:0	0.17 ± 0.00	2.24 ± 0.12	0.82 ± 0.18	1.13
C18:0	1.35 ± 0.00	7.23 ± 0.18	9.30 ± 0.13	0.03
C19:0	-	0.39 ± 0.02	0.45 ± 0.04	0.48
C20:0	-	0.25 ± 0.00	1.03 ± 0.01	-
C21:0	0.03 ± 0.00	-	-	-
C22:0	0.02 ± 0.00	-	0.72 ± 0.02	-
C24:0	0.08 ± 0.00	-	-	-
C16:1n-7	0.65 ± 0.00	3.46 ± 0.61	1.84 ± 0.03	-
C18:1n-7	-	2.53 ± 0.24	2.43 ± 0.04	5.82
C18:1n-9	2.63 ± 0.02	20.26 ± 0.53	19.63 ± 0.33	29.26
C20:1	0.10 ± 0.00	1.54 ± 0.19	1.53 ± 0.03	-
C18:3n-3	0.99 ± 0.00	-	3.07 ± 0.21	-
C20:3n-3	0.11 ± 0.00	-	-	-
C20:5n-3 (EPA)	3.21 ± 0.00	15.17 ± 0.63	13.99 ± 0.14	-
C22:6n-3 (DHA)	2.19 ± 0.00	4.94 ± 0.67	9.93 ± 0.15	-
C18:2n-6c	1.57 ± 0.00	7.52 ± 0.20	14.17 ± 0.22	-
C18:3n-6	0.15 ± 0.00	-	-	-
C20:2n-6	0.19 ± 0.00	1.14 ± 0.02	1.69 ± 0.05	-
C20:3n-6	0.04 ± 0.00	0.73 ± 0.08	-	-
C20:4n-6	0.09 ± 0.00	10.38 ± 0.03	2.29 ± 0.03	-

## Data Availability

No new data were created or analyzed in this study. Data sharing is not applicable to this article.

## References

[1] Oficialdegui F.J., Sánchez M.I., Clavero M. (2020). One century away from home: How the red swamp crayfish took over the world. Rev. Fish Biol. Fish..

[2] Berge G.M., Ruyter B., Åsgård T. (2004). Conjugated linoleic acid in diets for juvenile Atlantic salmon (*Salmo salar*); effects on fish performance, proximate composition, fatty acid and mineral content. Aquaculture.

[3] Tanakol R., Yazici Z., Şener E., Sencer E. (1999). Fatty acid composition of 19 species of fish from the Black Sea and the Marmara Sea. Lipids.

[4] El-Sherif S.A.E.-H., Abd El-Ghafour S. (2015). Nutritive value of canned River Nile Crayfish (*Procambarus clarkii*) products. Egypt. J. Aquat. Res..

[5] Wang J., Ye J., Zhang Z., An Z., Wang T., Dong X. (2023). Comparison of the nutrient value, nonspecific immunity, and intestinal microflora of red swamp crayfish (*Procambarus clarkii*) in different culture modes. Aquac. Rep..

[6] Li J., Huang J., Li C., Zhang Y., Wang Y., Hou S., Cheng Y., Li J. (2021). Evaluation of the nutritional quality of edible tissues (muscle and hepatopancreas) of cultivated Procambarus clarkii using biofloc technology. Aquac. Rep..

[7] Zaglol N.F., Eltadawy F. (2009). Study on chemical quality and nutrition value of fresh water crayfish (*Procambarus clarkii*). J. Arab. Aquac. Soc..

[8] Farrag M.M., El-Geddawy M.-A.M., Ahmed Z.S. (2022). More evidences for the nutritional quality and future exploitation of the invasive crayfish Procambarus clarkii (Girard, 1852) from the River Nile, Egypt. Egypt. J. Aquat. Res..

[9] Lu X., Peng D., Chen X., Wu F., Jiang M., Tian J., Liu W., Yu L., Wen H., Wei K. (2020). Effects of dietary protein levels on growth, muscle composition, digestive enzymes activities, hemolymph biochemical indices and ovary development of pre-adult red swamp crayfish (*Procambarus clarkii*). Aquac. Rep..

[10] Zhang Y., Yang B., Yang L., Jiang S., Lu J., Lin L. (2023). Comparison of the nutritional qualities of the pond, rice-field and wild crayfish (*Procambarus clarkii*) meat. Food Chem. Adv..

[11] Amine T.M., Aiad A.S., Abu El-Nile M.O. (2008). Evaluation of chemical quality and nutrition value of fresh water cray fish (*Procambarus clarkii*). J. High Inst. Public Health.

[12] Bai S., Qin D., Chen Z., Wu S., Tang S., Gao L., Wang P. (2022). Geographic origin discrimination of red swamp crayfish *Procambarus clarkii* from different Chinese regions using mineral element analysis assisted by machine learning techniques. Food Control.

[13] Liu Q., Long Y., Li B., Zhao L., Luo J., Xu L., Luo W., Du Z., Zhou J., Yang S. (2020). Rice-shrimp culture: A better intestinal microbiota, immune enzymatic activities, and muscle relish of crayfish (*Procambarus clarkii*) in Sichuan Province. Appl. Microbiol. Biotechnol..

[14] Liu M., Chen C., Wu Q.-C., Chen J.-L., Dai L.-S., Chu S.H., Liu Q.-N. (2021). Chitinase involved in immune regulation by mediated the toll pathway of crustacea *Procambarus clarkii*. Fish Shellfish. Immunol..

[15] Qin Z., Babu V.S., Lin H., Dai Y., Kou H., Chen L., Li J., Zhao L., Lin L. (2019). The immune function of prophenoloxidase from red swamp crayfish (*Procambarus clarkii*) in response to bacterial infection. Fish Shellfish. Immunol..

[16] Meng Q., Chen J., Xu C., Huang Y., Wang Y., Wang T., Zhai X., Gu W., Wang W. (2013). The characterization, expression and activity analysis of superoxide dismutases (SODs) from *Procambarus clarkii*. Aquaculture.

[17] Yan N., Chen X. (2015). Sustainability: Don’t waste seafood waste. Nature.

[18] Du J., Tan E., Kim H.J., Zhang A., Bhattacharya R., Yarema K.J. (2014). Comparative evaluation of chitosan, cellulose acetate, and polyethersulfone nanofiber scaffolds for neural differentiation. Carbohydr. Polym..

[19] Hamed I., Özogul F., Regenstein J.M. (2016). Industrial applications of crustacean by-products (chitin, chitosan, and chitooligosaccharides): A review. Trends Food Sci. Technol..

[20] Parvez S., Rahman M.M., Khan M.A.H., Islam J.M.M., Ahmed M., Ahmed B. (2012). Preparation and characterization of artificial skin using chitosan and gelatin composites for potential biomedical application. Polym. Bull..

[21] Usmani Z., Sharma M., Awasthi A.K., Sivakumar N., Lukk T., Pecoraro L., Thakur V.K., Roberts D., Newbold J., Gupta V.K. (2021). Bioprocessing of waste biomass for sustainable product development and minimizing environmental impact. Bioresour. Technol..

[22] Shamshina J.L., Berton P., Rogers R.D. (2019). Advances in functional chitin materials: A review. ACS Sustain. Chem. Eng..

[23] Zhao T., Yan X., Sun L., Yang T., Hu X., He Z., Liu F., Liu X. (2019). Research progress on extraction, biological activities and delivery systems of natural astaxanthin. Trends Food Sci. Technol..

[24] Sui X., Yue R., Wang L., Han Y. (2015). Process Optimization of Astaxanthin Extraction from Antarctic kill (*Euphausia superba*) by subcritical R134a. Proceedings of the 3rd International Conference on Material, Mechanical and Manufacturing Engineering (IC3ME 2015).

[25] Yamashita E. (2013). Astaxanthin as a medical food. Funct. Foods Health Dis..

[26] Visioli F., Artaria C. (2017). Astaxanthin in cardiovascular health and disease: Mechanisms of action, therapeutic merits, and knowledge gaps. Food Funct..

[27] Lim K.C., Yusoff F.M., Shariff M., Kamarudin M.S. (2018). Astaxanthin as feed supplement in aquatic animals. Rev. Aquac..

[28] McCall B., McPartland C.K., Moore R., Frank-Kamenetskii A., Booth B.W. (2018). Effects of astaxanthin on the proliferation and migration of breast cancer cells in vitro. Antioxidants.

[29] Sun C., Xu W.-T., Zhang H.-W., Dong L.-P., Zhang T., Zhao X.-F., Wang J.-X. (2011). An anti-lipopolysaccharide factor from red swamp crayfish, *Procambarus clarkii*, exhibited antimicrobial activities in vitro and in vivo. Fish Shellfish. Immunol..

[30] Zhu J.-J., Ye Z.-Z., Li C.-S., Kausar S., Abbas M.N., Xiang G.-H., Qian X.-Y., Dai L.-S. (2019). Identification and molecular characterization of a novel anti-lipopolysaccharide factor (ALF) from red swamp crayfish, *Procambarus clarkii*. Int. J. Biol. Macromol..

[31] Shao Y., Xiong G., Ling J., Hu Y., Shi L., Qiao Y., Yu J., Cui Y., Liao L., Wu W. (2018). Effect of ultra-high pressure treatment on shucking and meat properties of red swamp crayfish (*Procambarus clarkia*). LWT.

[32] Shi L., Xiong G., Yin T., Ding A., Li X., Wu W., Qiao Y., Liao L., Jiao C., Wang L. (2020). Effects of ultra-high pressure treatment on the protein denaturation and water properties of red swamp crayfish (*Procambarus clarkia*). LWT.

[33] Wang L., Shi L., Jiao C., Qiao Y., Wu W., Li X., Wang J., Ding A., Liao L., Xiong G. (2020). Effect of ultrasound combined with ozone water pretreatment on the bacterial communities and the physicochemical properties of red swamp crayfish meat (*Procambarus clarkii*). Food Bioprocess Technol..

[34] Sun R., Xu W., Xiong L., Jiang N., Xia J., Zhu Y., Wang C., Liu Q., Ma Y., Luo H. (2023). The combined effects of ultrasound and plasma-activated water on microbial inactivation and quality attributes of crayfish during refrigerated storage. Ultrason. Sonochemistry.

[35] Jiang Q., Zhang J., Gao P., Yu D., Yang F., Xu Y., Xia W., Chen N., Jiao T. (2023). Effects of cooking temperature and time on physicochemical, textural, structural, and microbiological features of fresh crayfish (*Procambarus clarkii*). J. Food Meas. Charact..

[36] Yang B., Zhang Y., Jiang S., Lu J., Lin L. (2023). Effects of different cooking methods on the edible quality of crayfish (*Procambarus clarkii*) meat. Food Chem. Adv..

[37] Fan H., Fan D., Huang J., Zhao J., Yan B., Ma S., Zhou W., Zhang H. (2020). Cooking evaluation of crayfish (*Procambarus clarkia*) subjected to microwave and conduction heating: A visualized strategy to understand the heat-induced quality changes of food. Innov. Food Sci. Emerg. Technol..

[38] Lin D., Sun L.-C., Chen Y.-L., Liu G.-M., Miao S., Cao M.-J. (2022). Shrimp spoilage mechanisms and functional films/coatings used to maintain and monitor its quality during storage. Trends Food Sci. Technol..

[39] Huang J., Hu Z., Li G., Xiang Y., Chen J., Hu Y. (2022). Preservation mechanism of liquid nitrogen freezing on crayfish (*Procambarus clarkia*): Study on the modification effects in biochemical and structural properties. J. Food Process. Preserv..

[40] Shi L., Xiong G., Ding A., Li X., Wu W., Qiao Y., Liao L., Wang L. (2018). Effects of freezing temperature and frozen storage on the biochemical and physical properties of *Procambarus clarkii*. Int. J. Refrig..

[41] Çoban M.Z. (2021). Effectiveness of chitosan/propolis extract emulsion coating on refrigerated storage quality of crayfish meat (*Astacus leptodactylus*). CyTA-J. Food.

[42] Liu W., Shen Y., Li N., Mei J., Xie J. (2019). Application of gelatin incorporated with red pitaya peel methanol extract as edible coating for quality enhancement of crayfish (*Procambarus clarkii*) during refrigerated storage. J. Food Qual..

[43] Kandeepan G., Tahseen A. (2022). Modified atmosphere packaging (map) of meat and meat products: A review. J. Packag. Technol. Res..

[44] Cremades O., Álvarez-Ossorio C., Gutierrez-Gil J.F., Parrado J., Bautista J. (2011). Quality changes of cooked crayfish (*Procambarus clarkii*) tails without additives during storage under protective atmospheres. J. Food Process. Preserv..

[45] Pothuri P., Marshall D.L., Mcmillin K.W. (1996). Combined effects of packaging atmosphere and lactic acid on growth and survival of *Listeria monocytogenes* in crayfish tail meat at 4 °C. J. Food Prot..

[46] Zhu R., Xiong Z., Li D. (2023). Complex organic acids treatment on crayfish (*Procambarus clarkii*): The relationship between myofibrillar protein structure and meat quality. Int. J. Food Sci. Technol..

[47] Wei B., Gao Y., Zheng Y., Yu J., Fu X., Bao H., Guo Q., Hu H. (2024). Changes in the Quality and Microbial Communities of Precooked Seasoned Crayfish Tail Treated with Microwave and Biological Preservatives during Room Temperature Storage. Foods.

[48] Guo C., Le Y., Lu Y., Yang H., He Y. (2024). Effect of oxygen supplement on post-mortem metabolic profile of shrimp during cold storage. Food Res. Int..

[49] Coates C.J., Söderhäll K. (2021). The stress–immunity axis in shellfish. J. Invertebr. Pathol..

[50] Huang Z., Guan W., Lyu X., Chen R., Wu Y., Zheng G., Mao L. (2023). Impacts of long-time transportation on whiteleg shrimp (*Penaeus vannamei*) muscle quality and underlying biochemical mechanisms. J. Sci. Food Agric..

[51] Mota V.C., Siikavuopio S.I., James P. (2021). Physiological responses to live air transport of red king crab (*Paralithodes camtschaticus*). Fish. Res..

[52] Özturan S., Ünal Şengör G.F. (2022). Effects of cooking methods on the quality and safety of crayfish (*Astacus leptodactylus* Eschscholtz, 1823) during chilled storage. J. Food Process. Preserv..

[53] Han J., Sun Y., Zhang T., Wang C., Xiong L., Ma Y., Zhu Y., Gao R., Wang L., Jiang N. (2023). The preservable effects of ultrasound-assisted alginate oligosaccharide soaking on cooked crayfish subjected to Freeze-Thaw cycles. Ultrason. Sonochemistry.

[54] Sun Y., Zhang M., Bhandari B., Yang C.-H. (2019). Ultrasound treatment of frozen crayfish with chitosan Nano-composite water-retaining agent: Influence on cryopreservation and storage qualities. Food Res. Int..

[55] Arvanitoyannis I.S., Kassaveti A. (2008). Fish industry waste: Treatments, environmental impacts, current and potential uses. Int. J. Food Sci. Technol..

[56] Yan B., Yang H., Zhang N., Cheng J., Huang J., Zhao J., Zhang H., Chen W., Fan D. (2023). Microwave-assisted depolymerization of chitin and chitosan extracted from crayfish shells waste: A sustainable approach based on graphene oxide catalysis. Int. J. Biol. Macromol..

[57] Rodrigo-Baños M., Garbayo I., Vílchez C., Bonete M.J., Martínez-Espinosa R.M. (2015). Carotenoids from Haloarchaea and their potential in biotechnology. Mar. Drugs.

[58] Maciassanchez M., Mantell C., Rodríguez M., Martinezdelaossa E., Lubián L., Montero O., Sánchez M., de la Ossa E.M. (2009). Comparison of supercritical fluid and ultrasound-assisted extraction of carotenoids and chlorophyll a from *Dunaliella salina*. Talanta.

[59] Sachindra N., Bhaskar N., Mahendrakar N. (2006). Recovery of carotenoids from shrimp waste in organic solvents. Waste Manag..

[60] Bi W., Tian M., Zhou J., Row K.H. (2010). Task-specific ionic liquid-assisted extraction and separation of astaxanthin from shrimp waste. J. Chromatogr. B.

[61] Irna C., Jaswir I., Othman R., Jimat D.N. (2018). Comparison between high-pressure processing and chemical extraction: Astaxanthin yield from six species of shrimp carapace. J. Diet. Suppl..

[62] Deng J.-J., Mao H.-H., Fang W., Li Z.-Q., Shi D., Li Z.-W., Zhou T., Luo X.-C. (2020). Enzymatic conversion and recovery of protein, chitin, and astaxanthin from shrimp shell waste. J. Clean. Prod..

[63] Wang L., Hu J., Lv W., Lu W., Pei D., Lv Y., Wang W., Zhang M., Ding R., Lv M. (2021). Optimized extraction of astaxanthin from shrimp shells treated by biological enzyme and its separation and purification using macroporous resin. Food Chem..

[64] Hamdi S., Elsayed N., Algayar M., Ishak V., Ahmed M., Ahmed S., Kamal M., Abd El-Ghany M. (2022). Biological extraction, HPLC quantification and medical applications of astaxanthin extracted from crawfish “*Procambarus clarkii*” exoskeleton by-product. Biology.

[65] Su F., Yu W., Liu J. (2020). Comparison of effect of dietary supplementation with *Haematococcus pluvialis* powder and synthetic astaxanthin on carotenoid composition, concentration, esterification degree and astaxanthin isomers in ovaries, hepatopancreas, carapace, epithelium of adult female Chinese mitten crab (*Eriocheir sinensis*). Aquaculture.

[66] Stachowiak B., Szulc P. (2021). Astaxanthin for the food industry. Molecules.

[67] Lim K.C., Yusoff F.M., Shariff M., Kamarudin M.S. (2019). Dietary administration of astaxanthin improves feed utilization, growth performance and survival of Asian seabass, *Lates calcarifer* (Bloch, 1790). Aquac. Nutr..

[68] Hansen, (2016). J.; Puvanendran, V.; Bangera, R. Broodstock diet with water and astaxanthin improve condition and egg output of brood fish and larval survival in Atlantic cod, *Gadus morhua* L. Aquac. Res..

[69] Zhu X., Hao R., Zhang J., Tian C., Hong Y., Zhu C., Li G. (2022). Dietary astaxanthin improves the antioxidant capacity, immunity and disease resistance of coral trout (*Plectropomus leopardus*). Fish Shellfish. Immunol..

[70] Hossain A.K.M.M., Brennan M.A., Mason S.L., Guo X., Zeng X.A., Brennan C.S. (2017). The effect of astaxanthin-rich microalgae “*Haematococcus pluvialis*” and wholemeal flours incorporation in improving the physical and functional properties of cookies. Foods.

[71] Wang H., He W., Dansou D.M., Zhang H., Nugroho R.D., Tang C., Guo X., Yu Y., Zhao Q., Qin Y. (2022). Astaxanthin improved the storage stability of docosahexaenoic acid-enriched eggs by inhibiting oxidation of non-esterified poly-unsaturated fatty acids. Food Chem..

[72] Montero P., Calvo M., Gómez-Guillén M., Gómez-Estaca J. (2016). Microcapsules containing astaxanthin from shrimp waste as potential food coloring and functional ingredient: Characterization, stability, and bioaccessibility. LWT.

[73] Bhuvaneswari S., Arunkumar E., Viswanathan P., Anuradha C.V. (2010). Astaxanthin restricts weight gain, promotes insulin sensitivity and curtails fatty liver disease in mice fed a obesity-promoting diet. Process Biochem..

[74] Otsuka T., Shimazawa M., Inoue Y., Nakano Y., Ojino K., Izawa H., Tsuruma K., Ishibashi T., Hara H. (2016). Astaxanthin protects against retinal damage: Evidence from in vivo and in vitro retinal ischemia and reperfusion models. Curr. Eye Res..

[75] Masoudi A., Dargahi L., Abbaszadeh F., Pourgholami M.H., Asgari A., Manoochehri M., Jorjani M. (2017). Neuroprotective effects of astaxanthin in a rat model of spinal cord injury. Behav. Brain Res..

[76] Zhang X.S., Zhang X., Wu Q., Li W., Wang C.X., Xie G.B., Zhou X.M., Shi J.X., Zhou M.L. (2014). Astaxanthin offers neuroprotection and reduces neuroinflammation in experimental subarachnoid hemorrhage. J. Surg. Res..

[77] Hu J., Lu W., Lv M., Wang Y., Ding R., Wang L. (2019). Extraction and purification of astaxanthin from shrimp shells and the effects of different treatments on its content. Rev. Bras. Farmacogn..

[78] Sachindra N., Mahendrakar N. (2005). Process optimization for extraction of carotenoids from shrimp waste with vegetable oils. Bioresour. Technol..

[79] Pu J., Sathivel S. (2011). Kinetics of lipid oxidation and degradation of flaxseed oil containing crawfish (*Procambarus clarkii*) astaxanthin. J. Am. Oil Chem. Soc..

[80] Charest D.J., Balaban M.O., Marshall M.R., Cornell J.A. (2001). Astaxanthin extraction from crawfish shells by supercritical CO_2_ with ethanol as cosolvent. J. Aquat. Food Prod. Technol..

[81] Pachapur V.L., Guemiza K., Rouissi T., Sarma S.J., Brar S.K. (2016). Novel biological and chemical methods of chitin extraction from crustacean waste using saline water. J. Chem. Technol. Biotechnol..

[82] Sedaghat F., Yousefzadi M., Toiserkani H., Najafipour S. (2017). Bioconversion of shrimp waste *Penaeus merguiensis* using lactic acid fermentation: An alternative procedure for chemical extraction of chitin and chitosan. Int. J. Biol. Macromol..

[83] Pakizeh M., Moradi A., Ghassemi T. (2021). Chemical extraction and modification of chitin and chitosan from shrimp shells. Eur. Polym. J..

[84] Kaya M., Baran T., Karaarslan M. (2015). A new method for fast chitin extraction from shells of crab, crayfish and shrimp. Nat. Prod. Res..

[85] Dun Y., Li Y., Xu J., Hu Y., Zhang C., Liang Y., Zhao S. (2019). Simultaneous fermentation and hydrolysis to extract chitin from crayfish shell waste. Int. J. Biol. Macromol..

[86] Kumari S., Annamareddy S.H.K., Abanti S., Rath P.K. (2017). Physicochemical properties and characterization of chitosan synthesized from fish scales, crab and shrimp shells. Int. J. Biol. Macromol..

[87] El-Naggar M.M., Abou-Elmagd W.S.I., Suloma A., El-Shabaka H.A., Khalil M.T., El-Rahman F.A.A. (2019). Optimization and physicochemical characterization of chitosan and chitosan nanoparticles extracted from the crayfish *Procambarus clarkii* wastes. J. Shellfish. Res..

[88] Bajaj M., Winter J., Gallert C. (2011). Effect of deproteination and deacetylation conditions on viscosity of chitin and chitosan extracted from *Crangon crangon* shrimp waste. Biochem. Eng. J..

[89] Guo N., Sun J., Zhang Z., Mao X. (2019). Recovery of chitin and protein from shrimp head waste by endogenous enzyme autolysis and fermentation. J. Ocean. Univ. China.

[90] Sjaifullah A., Santoso A.B. (2016). Autolytic isolation of chitin from white shrimp (*Penaues vannamei*) waste. Procedia Chem..

[91] Liu P., Liu S., Guo N., Mao X., Lin H., Xue C., Wei D. (2014). Cofermentation of *Bacillus licheniformis* and *Gluconobacter oxydans* for chitin extraction from shrimp waste. Biochem. Eng. J..

[92] Sixto-Berrocal A.M., Vázquez-Aldana M., Miranda-Castro S.P., Martínez-Trujillo M.A., Cruz-Díaz M.R. (2023). Chitin/chitosan extraction from shrimp shell waste by a completely biotechnological process. Int. J. Biol. Macromol..

[93] Ghorbel-Bellaaj O., Younes I., Maâlej H., Hajji S., Nasri M. (2012). Chitin extraction from shrimp shell waste using *Bacillus bacteria*. Int. J. Biol. Macromol..

[94] Abdou E.S., Nagy K.S., Elsabee M.Z. (2008). Extraction and characterization of chitin and chitosan from local sources. Bioresour. Technol..

[95] Guo S., Ren Y., Chang R., He Y., Zhang D., Guan F., Yao M. (2022). Injectable self-healing adhesive chitosan hydrogel with antioxidative, antibacterial, and hemostatic activities for rapid hemostasis and skin wound healing. ACS Appl. Mater. Interfaces.

[96] Abbas M., Hussain T., Arshad M., Ansari A.R., Irshad A., Nisar J., Hussain F., Masood N., Nazir A., Iqbal M. (2019). Wound healing potential of curcumin cross-linked chitosan/polyvinyl alcohol. Int. J. Biol. Macromol..

[97] Chen C.-Y., Chung Y.-C. (2012). Antibacterial effect of water-soluble chitosan on representative dental pathogens *Streptococcus mutans* and *Lactobacilli brevis*. J. Appl. Oral Sci..

[98] Altaher H. (2012). The use of chitosan as a coagulant in the pre-treatment of turbid sea water. J. Hazard. Mater..

[99] Liaw B.-S., Chang T.-T., Chang H.-K., Liu W.-K., Chen P.-Y. (2020). Fish scale-extracted hydroxyapatite/chitosan composite scaffolds fabricated by freeze casting—An innovative strategy for water treatment. J. Hazard. Mater..

[100] Duran A., Kahve H.I. (2020). The effect of chitosan coating and vacuum packaging on the microbiological and chemical properties of beef. Meat Sci..

[101] Gassara F., Antzak C., Ajila C.M., Sarma S.J., Brar S.K., Verma M. (2015). Chitin and chitosan as natural flocculants for beer clarification. J. Food Eng..

[102] Achmad H., Ramadhany Y.F. (2017). Effectiveness of chitosan tooth paste from white shrimp (*Litopenaeusvannamei*) to reduce number of *Streptococcus mutans* in the case of early childhood caries. J. Int. Dent. Med. Res..

[103] Ta Q., Ting J., Harwood S., Browning N., Simm A., Ross K., Olier I., Al-Kassas R. (2021). Chitosan nanoparticles for enhancing drugs and cosmetic components penetration through the skin. Eur. J. Pharm. Sci..

[104] Peng F., Li J., Gong Z., Yue B., Wang X., Manyande A., Du H. (2022). Investigation of bioaccumulation and human health risk assessment of heavy metals in crayfish (*Procambarus clarkii*) farming with a rice-crayfish-based coculture breeding modes. Foods.

[105] Niño S.A., Morales-Martínez A., Chi-Ahumada E., Carrizales L., Salgado-Delgado R., Pérez-Severiano F., Díaz-Cintra S., Jiménez-Capdeville M.E., Zarazua S. (2018). Arsenic exposure contributes to the bioenergetic damage in an Alzheimer’s disease model. ACS Chem. Neurosci..

[106] Aguilera-Velázquez J.R., Calleja A., Moreno I., Bautista J., Alonso E. (2023). Metal profiles and health risk assessment of the most consumed rice varieties in Spain. J. Food Compos. Anal..

[107] Mo A., Huang Y., Gu Z., Liu C., Wang J., Yuan Y. (2022). Health risk assessment and bioaccumulation of heavy metals in *Procambarus clarkii* from six provinces of China. Environ. Sci. Pollut. Res..

[108] Zhang Y., Li Z., Kholodkevich S., Sharov A., Chen C., Feng Y., Ren N., Sun K. (2020). Effects of cadmium on intestinal histology and microbiota in freshwater crayfish (*Procambarus clarkii*). Chemosphere.

[109] Kouba A., Buřič M., Kozák P. (2010). Bioaccumulation and effects of heavy metals in crayfish: A review. Water Air Soil Pollut..

[110] Xiong B., Xu T., Li R., Johnson D., Ren D., Liu H., Xi Y., Huang Y. (2020). Heavy metal accumulation and health risk assessment of crayfish collected from cultivated and uncultivated ponds in the Middle Reach of Yangtze River. Sci. Total Environ..

[111] Tan Y., Peng B., Wu Y., Xiong L., Sun J., Peng G., Bai X. (2021). Human health risk assessment of toxic heavy metal and metalloid intake via consumption of red swamp crayfish (*Procambarus clarkii*) from rice-crayfish co-culture fields in China. Food Control.

[112] Hong Y., Huang Y., Yan G., Yin H., Huang Z. (2021). DNA damage, immunotoxicity, and neurotoxicity induced by deltamethrin on the freshwater crayfish, *Procambarus clarkii*. Environ. Toxicol..

[113] Wu N., Wei H., Shen H., Wu T.T., Guo M. (2012). Acute toxic effects of deltamethrin on red swamp crayfish, *Procambarus clarkii* (Decapoda, Cambaridae). Crustaceana.

[114] Zhang Y., Li Z., Zhang Y., Sun K., Ren N., Li M. (2022). Acute toxic effects of diclofenac exposure on freshwater crayfish (*Procambarus clarkii*): Insights from hepatopancreatic pathology, molecular regulation and intestinal microbiota. Ecotoxicol. Environ. Saf..

[115] Lehrer S., Ayuso R., Reese G. (2003). Seafood allergy and allergens: A review. Mar. Biotechnol..

[116] Ruethers T., Taki A.C., Johnston E., Nugraha R., Le T.T.K., Kalic T., McLean T., Kamath S.D., Lopata A.L. (2018). Seafood allergy: A comprehensive review of fish and shellfish allergens. Mol. Immunol..

[117] Chen H.-L., Mao H.-Y., Cao M.-J., Cai Q.-F., Su W.-J., Zhang Y.-X., Liu G.-M. (2013). Purification, physicochemical and immunological characterization of arginine kinase, an allergen of crayfish (*Procambarus clarkii*). Food Chem. Toxicol..

[118] Chen H.-L., Cao M.-J., Cai Q.-F., Su W.-J., Mao H.-Y., Liu G.-M. (2013). Purification and characterisation of sarcoplasmic calcium-binding protein, a novel allergen of red swamp crayfish (*Procambarus clarkii*). Food Chem..

[119] Zhang Y.X., Chen H.L., Maleki S.J., Cao M.J., Zhang L.J., Su W.J., Liu G.M. (2015). Purification, characterization, and analysis of the allergenic properties of myosin light chain in *Procambarus clarkii*. J. Agric. Food Chem..

[120] Yang Y., Yan H.-F., Zhang Y.-X., Chen H.-L., Cao M.-J., Li M.-S., Zhang M.-L., He X.-R., Liu G.-M. (2020). Expression and epitope identification of myosin light chain isoform 1, an allergen in *Procambarus clarkii*. Food Chem..

[121] Faisal M., Buckow R., Vasiljevic T., Donkor O. (2019). Effect of simulated digestion on antigenicity of banana prawn (*Fenneropenaeus merguiensis*) after high pressure processing at different temperatures. Food Control.

[122] Zhang Z., Zhang X., Chen W., Zhou P. (2018). Conformation stability, in vitro digestibility and allergenicity of tropomyosin from shrimp (*Exopalaemon modestus*) as affected by high intensity ultrasound. Food Chem..

[123] Muanghorn W., Konsue N., Sham H., Othman Z., Mohamed F., Noor N.M., Othman N., Akmal N.S.S.M.N., Fauzi N.A., Solomen M.M.P.D. (2018). Effects of gamma irradiation on tropomyosin allergen, proximate composition and mineral elements in giant freshwater prawn (*Macrobrachium rosenbergii*). J. Food Sci. Technol..

[124] Mejrhit N., Azdad O., Chda A., El Kabbaoui M., Bousfiha A., Bencheikh R., Tazi A., Aarab L. (2017). Evaluation of the sensitivity of Moroccans to shrimp tropomyosin and effect of heating and enzymatic treatments. Food Agric. Immunol..

[125] Zhang Z., Zhao Y., Han Y., Yang B., Lin H., Li Z. (2022). The natural substances with anti-allergic properties in food allergy. Trends Food Sci. Technol..

[126] Liu G., Luo J., Xiong W., Meng T., Zhang X., Liu Y., Liu C., Che H. (2024). Chlorogenic acid alleviates crayfish allergy by altering the structure of crayfish tropomyosin and upregulating TLR8. Food Chem..

[127] Hobbs Iii H., Jass J.P., Huner J.V. (1989). A review of global crayfish introductions with particular emphasis on two North American species (Decapoda, Cambaridae). Crustaceana.

[128] Herrmann A., Grabow K., Martens A. (2022). The invasive crayfish Faxonius immunis causes the collapse of macroinvertebrate communities in Central European ponds. Aquat. Ecol..

[129] O’Hea Miller S.B., Davis A.R., Wong M.Y. (2024). The Impacts of Invasive Crayfish and Other Non-Native Species on Native Freshwater Crayfish: A Review. Biology.

[130] Manfrin C., Souty-Grosset C., Anastácio P.M., Reynolds J., Giulianini P.G. (2019). Detection and control of invasive freshwater crayfish: From traditional to innovative methods. Diversity.

